# The characteristics of tinnitus in workers exposed to noise

**DOI:** 10.1016/S1808-8694(15)30825-9

**Published:** 2015-10-18

**Authors:** Luciara Giacobe Steinmetz, Bianca Simone Zeigelboim, Adriana Bender Lacerda, Thais Catalani Morata, Jair Mendes Marques

**Affiliations:** 1 MSc on Communication Disorders at Universidade Tuiuti do Paraná, clinical speech and hearing therapist.; 2 PhD, Coordinator of the Masters and Doctoral Program on Communication Disorders at Universidade Tuiuti do Paraná.; 3 MSc on Communication Disorders at Universidade Tuiuti do Paraná, Professor at the undergraduate and specialization program on Clinical Audiology at Universidade Tuiuti do Paraná.; 4 Post-Doctorate at NIOSH - Professor - Communication Disorders Graduate Program - Universidade Tuiuti do Paraná and Researcher at the National Institute for Occupational Safety and Health, EUA; 5 PhD on Geodesic Sciences at Universidade Federal do Paraná, Professor at the Masters and Doctoral Program on Communication Disorders at Universidade Tuiuti do Paraná.; Institution: Universidade Tuiuti do Paraná. All authors have their curricula registered under the CNPq.

**Keywords:** hearing loss, prevention, occupational noise

## Abstract

Tinnitus is a common auditory complaint among individuals exposed to noise. **Aim:** this paper aims to study the characteristics of tinnitus in workers exposed to noise. **Study design:** this is a descriptive prospective study. Materials and method: Fifty-two individuals averaging 29 years of age were enrolled in a hearing loss prevention program at a meat processing plant. The participants were interviewed and had their hearing tested in 2005 and 2006. **Results:** seventy-one percent of the participants were found to have normal hearing. Tinnitus was present in 16% of the males and in 9% of the females. Mean noise exposure length was 7 years and noise levels ranged from 86 to 91 dBA (48%). Bilateral tinnitus (46%) of the hissing type (40%) and moderate intensity (49%) was the most prevalent. Symptoms began to be observed within one to five years after initial exposure to noise (67%) and manifested themselves in weekly episodes (41%) that bothered the patients mostly at night (34%). A significant correlation was observed between the frequency of tinnitus episodes and the noise levels to which workers were exposed. **Conclusion:** tinnitus should be included in hearing loss prevention programs in order to more comprehensively promote occupational hearing health.

## INTRODUCTION

Research indicates that approximately 17% of the population in general and 33% of the elderly population is troubled by tinnitus. It develops into its severe manifestation in 20% of the cases, leading to significant suffering in 4% of the population in general. Tinnitus may directly or indirectly impact individuals during labor and leisure, interfering in family and social relations, and in extreme cases resulting in suicide^1^, ^2^, ^3^, ^4^.

In Brazil, it is estimated that approximately 6 million people are affected by tinnitus. In spite of the many theories around the likely causes, there is no scientific proof due to the lack of objective, non-invasive methods to detect the condition and locate the associated neural activity^5^.

Tinnitus, regardless of hearing complaints, is an auditory symptom widely reported by individuals exposed to high sound pressure levels (SPL)^6^, ^7^, ^8^, ^9^. Studies have mentioned^7^ that prolonged exposure to occupational noise can not only lead to hearing loss, but also to tinnitus and hypoacusis. Excessive exposure to noise is the most important risk factor for hearing loss and tinnitus, followed by age and gender^10^. Authors^11^ have described duration of exposure and noise severity as reported by workers exposed to high SPL as significantly associated with tinnitus.

This study aims to study the characteristics of tinnitus reported by individuals exposed to occupational noise.

## MATERIALS AND METHOD

This paper was approved by the Institutional Ethics Committee at Universidade Tuiuti do Paraná under permit 063/2006. All participants read and signed free informed consent agreements.

Potential participants were sent a questionnaire as part of a hearing loss prevention program organized by company Frigorífica Volátil in 2005. The company had 372 employees, 120 females and 252 males. The questionnaire was handed at the beginning of a training program on hearing loss prevention to all employees so as to collect data on their hearing, professional, and personal lives. Two of the questions made reference to tinnitus and the side of its manifestation. Eighty-two individuals (22%) complained of tinnitus and were selected for screening.

Out of the 82 people originally screened for tinnitus, 20 (24%) had been fired, 5 (6%) claimed not to have tinnitus anymore, and 5 (6%) were on vacation at the time of the study. Fifty-two individuals were enrolled in the study.

After selecting the participants, we analyzed their periodic occupational hearing tests. Threshold tone audiometry tests were conducted in the company in 2005 and 2006, according to the criteria defined by Ordinance 19 from the Ministry of Labor^12^ as described below.

a) Participants were interviewed and demographic, medical history and labor history data were collected, along with tinnitus-related facts.
b) Participants had their acoustic meatuses inspected for earwax.
c) Threshold tone audiometry tests were carried out in a soundproof booth after participants had acoustically rested for 14 hours. We used an AD 229 Interacoustics audiometer with TDH-39 earphones calibrated in accordance with international standards (ISO 8253) to perform the tests. Air conduction audiometry was done for frequencies ranging from 250 Hz to 8 kHz, and when the threshold was above 25 dB the analysis was carried out via bone conduction audiometry using frequencies from 500 Hz to 4 kHz.

Audiometry tests were ranked in accordance with Ordinance 19 from the Ministry of Labor^12^. This ordinance covers preventive measures and states that the acceptable limits are those in which the audiograms show hearing thresholds equal to or lower than 25 dB (HL), for all examined frequencies. Findings of upper hearing thresholds above 25 dB (HL) at frequencies of 3 kHz and/or 4 kHz and/or 6 kHz in both air and bone conduction audiometry are indicative of noise-induced hearing loss (NIHL) in one or both sides. Audiograms not meeting the above mentioned criteria are not suggestive of NIHL.

Participants were then asked to answer the Brazilian Portuguese translated and adapted version of the Tinnitus Handicap Inventory (THI) questionnaire designed by Newman et al^13^. The THI consists of 25 questions grouped into three subcategories with three options to choose from for each question. Scores are assigned to the answers as follows: each ’yes’ is worth four points; each ’sometimes’ is worth two points; ’no’ answers are not given points. All points are then added together to assess tinnitus severity for each individual. Studies^14^, ^15^ suggest that the outcomes be divided into five degrees of severity, namely: Grade 1 - negligible tinnitus; Grade 2 - mild tinnitus; Grade 3 -moderate tinnitus; Grade 4 - severe tinnitus; and Grade 5 - catastrophic tinnitus. According to the literature^16^, tinnitus severity can be assessed the following way: Grade 1 tinnitus is only perceived in quiet environments; Grade 2 can be easily masked by background noise and easily forgotten when the subject is busy; Grade 3 tinnitus is heard in the presence of background noise and everyday tasks can still be performed in spite of it; Grade 4 tinnitus is almost always present, the subject’s sleep is disturbed and his or her daily life may be compromised: Grade 5 tinnitus is heard all the time, the subject’s sleep is altered, and some daily tasks may become difficult to perform.

### Statistical analysis

Descriptive analysis was done based on the following data collected from the interviews:

a) outcomes from medical, hearing, and professional history;
b) tinnitus characteristics in terms of side, duration (in years), frequency, duration of each episode, acoustic traits (low, high, hissing etc), intensity and period of the day when tinnitus is more disturbing. Using a 5% statistical significance level and Spearman’s correlation ratio, the following correlations were drawn in connection to tinnitus periodicity:
  a) noise level;
  b) time for which noise is experienced at work;
  c) audiometry results (normal x altered). After that, considering the 5% significance level through the chi-square test, the following correlations were drawn in relation to tinnitus severity:
    a) noise level;
    b) time for which noise is experienced at work;
    c) audiometry results (normal x altered).

## RESULTS

a) Interview results:

The age average of the studied population was 29 years. The interview and audiometry data from 52 individuals complaining of tinnitus was analyzed. Eleven of them were females (9% of the 120 female workers with the company) and 41 were males (16% of the 252 workers with the company).

**Graph 1:**
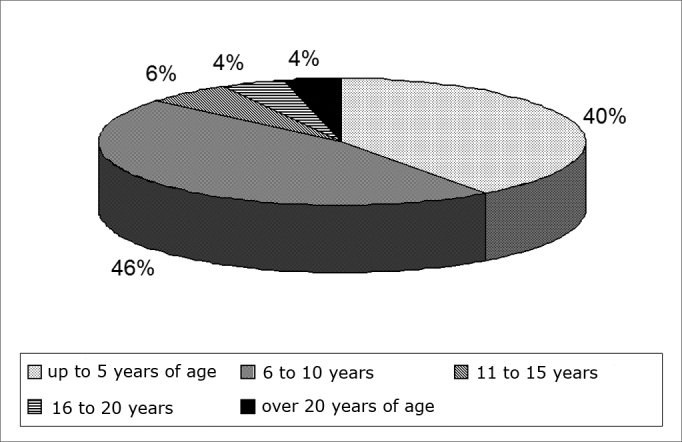
Percentage of subjects in relation to time of exposure to noise - not available

Forty-six percent of the population has been exposed to occupational noise for 6 to 10 years, as seen on Graph 1.

**Graph 2:**
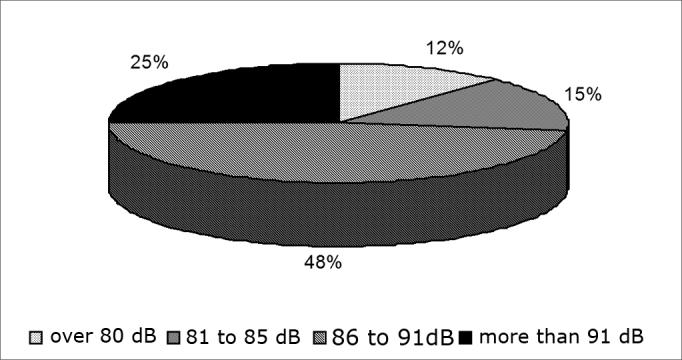
Percentage of subjects in relation to dose of noise exposure - dB - decibel

As far as noise level is concerned, 48% of the population is exposed to 86 to 91 dBA, as seen on Graph 2.

Table 1 shows the various findings connected to health, hearing, and medical history reported by the studied population. Still on hearing history, participants were asked if occupational noise had ever harmed their hearing. Fifteen (28%) subjects said that was the case.

**Table 1:** Number and percentage of individuals reporting health problems, hearing and medical history

Health Problems	YES		NO		UNKNOWN	
	N1	%	N2	%	N3	%
Vascular Changes	17	30	29	55	6	15
Metabolic Changes	4	8	40	77	8	15
Neurologic Changes	3	6	49	94	0	0
Dental Factors	18	35	33	63	1	2
Headache	12	23	39	75	1	2
Sinusitis	19	37	29	55	4	8

Hearing History Problems	YES		NO		UNKNOWN	

N1	%	N2	%	N3	%

Blast-related hearing loss	3	6	49	94	0	0
Hard hearing	21	40	31	60	0	0
Sensation of pressure in the ears	17	33	35	67	0	0
Chronic ear conditions	10	19	42	81	0	0
Mastoiditis	3	6	47	90	2	4
Vertigo	23	45	29	55	0	0
Hyperacusis	26	50	26	50	0	0
Ear surgery	0	0	52	100	0	0

Medical History	YES		NO		DID NOT HAVE THE HABIT	

N1	%	N2	%	N3	%

Medication	38	73	13	25	1	2
Smoking	10	19	33	64	9	17
Alcohol	3	6	49	94	0	0
Coffee/tea	37	71	15	29	0	0

N= number of subjects

b) Audiological evaluation results

**Figure 1:**
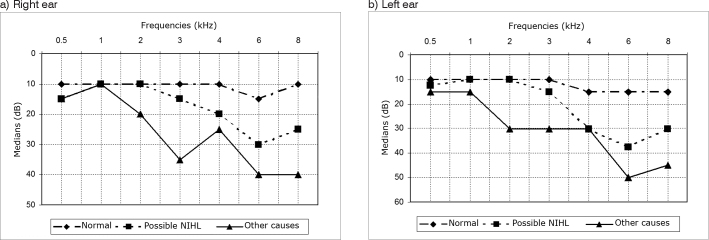
Group threshold medians indicative of NIHL, normal, and various causes (right and left ear)- not available

In terms of audiological findings, no alterations were found in the subjects’ acoustic meatuses. According to audiometry, 71% of the subjects had normal hearing, 12% had audiometric findings consistent with NIHL, and 17% had audiometric findings of hearing loss unrelated to noise. Median hearing thresholds were calculated for each of the ears of each individual. Figure 1 shows the median values for right and left ears respectively.

c) Tinnitus characterization

Table 2 shows the information related to tinnitus characterization.

**Table 2:** Tinnitus characterization

TINNITUS CHARACTERIZATION													
Age at onset		Time to onset in years		Side		Characteristic		Intensity		Periodicity		Time of day of greater distress	
Ranges	%	Ranges	%	Ear	%	Grades	%	Grades	%	Frequency	%	Frequency	%
12 - 32	65	£ 1	19	Right	21	Severe	8	High	13	Daily	35	All day	4
33 - 42	21	1 - 5	67	Left	29	High	29	Medium	49	Weekly	41	Morning	19
^3^ 42	2	6 - 10	12	Both	46	Hissing	40	Low	38	Bi-weekly	10	Afternoon	29
Does not know	12	3 10	2	Head	4	Whistle	19			Monthly	4	Evening	34
					Others	2			Sporadic	10	More than one time of the day	10	
					Does not know	2					Does not know	4	

d) Tinnitus severity

**Graph 3:**
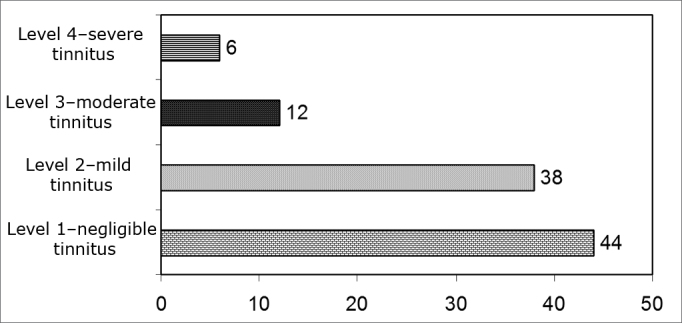
Individual percentage analysis of THI in relation to tinnitus grade - not available

Graph 3 shows the results on tinnitus severity degree.

e) Tinnitus correlations

Table 3 shows the results on the correlations between tinnitus periodicity and severity, and occupational noise level and time of exposure. When considering a 5% significance level, Spearman’s correlation ratio indicates the existence of a significant relationship between tinnitus periodicity and noise level to which the workers are exposed.

**Table 3:** Correlation between tinnitus severity, periodicity, noise level and time of exposure at work

VARIABLES	R	P
Tinnitus severity vs. Noise level	-0.096951	0.494130
Tinnitus severity vs. Time at company	0.052930	0.709396
Tinnitus periodicity vs. Noise level	0.284841	0.040688*
Tinnitus periodicity vs. Time at company	0.161259	0.253421

R = Spearman’s correlation ratio; P= significance level at 5%

The results concerning the correlation between tinnitus periodicity and audiometry findings can be seen on Table 4. When considering a 5% significance level, we can see through the chi-square test that p=0.9777, i.e., there is no significant dependence between audiometry findings and tinnitus periodicity.

**Table 4:** Correlations between tinnitus grade and audiometric test findings (normal x altered).

TINNITUS GRADE	AUDIOMETRIC TEST RESULTS	
	Normal	Altered
Negligible to mild	31	12
Moderate to severe	6	3

Considering the 5% significance level, it can be seen through the chi-square test that p=0.9380, i.e., there is no statistically significant correlation between audiometric test results and tinnitus grade.

The results concerning the correlation between tinnitus severity and audiometry findings can be seen on Table 5. When considering a 5% significance level, we can see through the chi-square test that p=0.9380, i.e., there is no significant dependence between audiometry findings and tinnitus grade.

**Table 5:** Correlation between tinnitus periodicity and audiometric test results (normal x altered).

TINNITUS PERIODICITY	AUDIOMETRIC TEST RESULTS	
	Normal	Altered
Daily or weekly	28	12
Bi-weekly, monthly, or sporadic	9	3

Considering the 5% significance level, it can be seen through the chi-square test that p=0.9777, i.e., there is no statistically significant correlation between audiometric test results and tinnitus periodicity.

## DISCUSSION

The research questionnaire was answered by 372 company employees during a hearing loss prevention program. Eighty-two (22%) complained of tinnitus. The author^8^ looked into 200 workers exposed to noise and observed that 15% of them had tinnitus. Other authors^17^ have found higher percentages of individuals suffering from tinnitus (48%). This difference in percentages may occur due to the fact that the populations studied by other authors come from companies where workers have been exposed to noise for longer, as opposed to 6.8 years on average as found in this study.

Many are the risk factors connected to tinnitus. Among them are: age, gender, diseases (ear, metabolic and neurologic diseases, vascular disorders, and dental factors), hearing loss, exposure to noise, ototoxic drugs, caffeine, nicotine, and alcohol^10^, ^11^, ^18^, ^19^. We tried therefore to learn about other risk factors for tinnitus aside from noise that the studied population could be exposed to.

Tinnitus was more prevalent in males (16%) than females (9%), as also seen in the literature^13^, ^20^, ^21^. The average age (29 years) of the studied population is lower than that of the literature, which ranged between 40 and 50 years^1^, ^16^, ^22^, ^23^.

Various studies suggest hearing status is an important factor associated with tinnitus prevalence in individuals exposed to noise^8^, ^11^, ^24^, ^25^, ^26^. The author^8^ concluded that 33% of the individuals with NIHL had tinnitus, whereas prevalence rates in groups of people with other hearing disorders and normal hearing were of 9.7% and 20% respectively.

Seventy-one percent of the participants in this study had normal audiometric findings. No statistically significant association was found between audiometry test results and tinnitus periodicity (p=0.9777) as or audiometry test results and tinnitus grade (p=0.9380) (see Tables 4 and 5). Tinnitus could be the first sign of exposure to intense noise and the symptom indicative of temporary hearing disorder^9^. There was no information on the prevalence of temporary hearing disorders in this population, but complaints of tinnitus deserve deeper investigation and follow-up.

The differences between the results observed in this study and in the literature in general concerning average ages and individual audiological findings may be explained by the fact that the population targeted by this analysis is younger (29 years of average age), has been exposed to noise for a shorter period of time (6.8 years on average), and has historically had fewer cases of hearing impairment.

The most frequently reported chronic health problems (Table 1) were sinusitis (37%), dental factors (35%), and vascular disorders (30%). Many medical history findings bear significant association with tinnitus. Neck and middle ear injuries, sinusitis, and headache increase the risk of tinnitus by 28% to 35%^11^.

Other significant risk factors for tinnitus are high blood lipid levels, high blood pressure, liver disorders, neck arthrosis, and alcohol abuse^27^.

Twenty-five percent of the studied population used medication. There are references in the literature^19^ to the side effects drugs such as acetylsalicylic acid, non-steroid anti-inflammatory drugs, antibiotics, aminoglycosides (mainly when administered concurrently with diuretics), sedatives, and tricyclic antidepressants may have in causing or worsening tinnitus (Table 1).

Sixty-four percent of the individuals enrolled in the study were non-smokers, 94% claimed not to drink alcohol on a daily basis, and 71% drink coffee or tea everyday (Table 1).

Caffeine and nicotine can worsen tinnitus as they act as stimulants and induce vasoconstriction^19^. According to the author, physicians believe that 50% of the patients complaining of tinnitus improve significantly when they cease to smoke or reduce caffeine intake. The author^28^ verified that in 84% of the studied patients alcohol consumption worsened tinnitus; 73% reported they were more aware of tinnitus after drinking; 49% stated tinnitus was worse the day after they had had alcohol; and 47% said they were sleepier. No differences were found on the alcohol-related side effects in relation to beverage type.

Average tinnitus onset time ranged between one and five years (Table 2). This finding is in agreement with other papers^29^, ^30^ that reported tinnitus onset within less than five years. Others^31^ have found that 50% of the studied population had tinnitus for over five years.

Bilateral tinnitus was more prevalent (46%), as seen in other papers^29^, ^30^, ^31^, ^32^. Other studies^33^ found that 28% of the individuals had unilateral tinnitus on the right ear, 36% had unilateral left-sided tinnitus, and 36% had bilateral tinnitus (Table 2).

Hissing (40%) was the most frequently reported type of tinnitus. Other authors found that cicada (35%), cricket (26%), hissing (26%), and pressure cooker-like (5.9%) tinnitus were among the most commonly reported types (Table 2).

Most participants complained of moderate intensity tinnitus (49%), as also seen in other studies^30^, ^31^ that found 29.4% to 92.9% of the individuals as having moderate to severe tinnitus (Table 2).

In terms of periodicity, most participants (41%) stated they had weekly episodes of tinnitus and that they were mostly bothered at night (34%) (Table 2). Statistically significant correlation was found between tinnitus periodicity and noise level (Table 3). This means that the individuals complaining of daily or weekly tinnitus are probably exposed to higher noise levels when compared to those complaining of bi-weekly, monthly, and sporadic episodes of tinnitus.

Participant responses concerning tinnitus severity varied from negligible (grade 1) to severe (grade 4). There were no cases of catastrophic tinnitus (grade 5). When studying the same case, the author^34^ verified that tinnitus interfered with the quality of life of workers exposed to noise, being the functional scale the most affected. This means those individuals are mostly impaired in their social daily tasks, while reading, sleeping, and when performing tasks that require concentration, hearing acuity, and attention. According to the data, tinnitus also increases tiredness and is accentuated with stress.

The data found in this study indicates that tinnitus should be included in hearing loss prevention programs, as it is a highly prevalent condition that may adversely impact various spheres of human life^35^, ^36^, ^37^.

Support groups for tinnitus carriers should be created with the following purposes:

1) provide advice (clarification and guidance on issues pertaining to tinnitus);
2) allow information and experience exchange between members; and
3) promote increased collective awareness over tinnitus.

Many authors^38^ have reported on the experience of having groups of tinnitus patients seen in hospitals and observed that group members developed new behaviors and learned how to cope better with the symptom.

Assessing the impact tinnitus has on the lives of workers and knowing its characteristics is possibly the first step to accepting the symptom, taking advice on it, following it up and managing the condition.

## CONCLUSION

Tinnitus affected 22% of the population analyzed in this study. Prevalence rates were higher among males (16%) and the sample was mostly made up by younger people (29 years old on average) with relatively little exposure to noise (6.8 years on average) at levels ranging from 86 and 91 dBA (48%). Seventy-one percent of the subjects with tinnitus had normal hearing.

Time to onset ranged between one and five years (67%) and age at onset varied between 12 and 32 years (65%). Bilateral tinnitus was more prevalent (46%), as well as tinnitus of the hissing type (40%) of moderate intensity (49%), occurring in weekly episodes (41%) and preferably at night (34%).

Workers with normal hearing (71%) should be included in hearing loss prevention programs for counseling and monitoring of the symptom so as to prevent hearing loss from settling in.
